# Cost-effectiveness analysis of sequential two-step screening versus direct colonoscopy screening for colorectal cancer: a large-scale survey in Eastern China

**DOI:** 10.3389/fonc.2025.1524172

**Published:** 2025-02-14

**Authors:** Yun Fu, Hao Li, Ao Xu, Zhongrong Yang, Peng Zhang, Weibing Wang

**Affiliations:** ^1^ Huzhou Center for Disease Control and Prevention, Huzhou, Zhejiang, China; ^2^ Zhongshan Hospital Affiliated to Fudan University, Shanghai, China; ^3^ School of Public Health, Fudan University, Shanghai, China

**Keywords:** cost-effectiveness, analysis, colorectal cancer, screening, survey

## Abstract

**Objectives:**

Despite the implementation of colorectal cancer (CRC) screening programs in many regions worldwide over the past few decades, the cost-effectiveness of these programs has been questioned owing to their acceptance rates. In this study, we evaluated the cost-effectiveness of screening strategies, quantified the impact of colonoscopy acceptance rates, and analyzed the underlying factors driving individual preferences.

**Methods:**

The cost-effectiveness of three strategies—no screening, sequential two-step screening (fecal immunochemical test and risk assessment, followed by colonoscopy), and colonoscopy screening—was evaluated from a societal perspective. This assessment was conducted using a decision-tree Markov model with the incremental cost-effectiveness ratio as the primary evaluation criterion.

**Results:**

Sequential screening was more cost-effective than colonoscopy screening (19,335 vs. 27,379 United States dollars per quality-adjusted life year). Ideal sequential screening could prevent 32.2%(691/2147) CRC deaths, whereas colonoscopy screening at the same colonoscopy acceptance rate (20.3%) could prevent 17.6%(377/2147) CRC deaths. When the acceptance rate of direct colonoscopy surpasses the threshold of 37.2%, the resulting health benefits likely outweigh those achieved using a the sequential two-step screening approach.

**Conclusions:**

Sequential screening is recommended for individuals in areas with constrained screening resources or during the early stages of regional screening program implementation. However, once screening habits are established, transitioning to direct colonoscopy screening becomes more favorable. Notably, reducing colonoscopy costs is the principal factor for enhancing an individual’s willingness to undergo the procedure.

## 1 Introduction

Globally, colorectal cancer (CRC) is the third most prevalent cancer and the second leading cause of cancer deaths (1). CRC also imposes a significant economic burden, ranking third among all cancers (2). Some patients with CRC present with typical symptoms, such as constipation, bloody stool, anorexia, nausea, and vomiting, whereas approximately 50% of these patients are asymptomatic (3). Thus, it is difficult for many patients with CRC to recognize the onset of the disease, with most presenting with advanced-stage disease and some even having metastasis (4). Patients with advanced CRC have a poor quality of life, unfavorable prognosis, and substantial treatment costs, such as chemotherapy (5–7). Therefore, reducing the incidence of CRC and increasing the rate of early diagnosis is vital.

Since 1975, large CRC screening programs have been launched in several countries, including the United States, some in Europe, Australia, and South Korea (8–11). Screening methods include guaiac fecal occult blood tests, fecal immunochemical tests (FITs), total colonoscopy, and flexible sigmoidoscopy (9). Nationwide screening programs have reduced the CRC incidence and CRC-specific mortality rates (12–14), whereas both rates have increased in regions lacking national screening programs, particularly in Asia (15, 16). More recently, however, colonoscopy screening programs have been reported to be ineffective in reducing CRC incidence and mortality rates (17).

Many countries have long-standing CRC screening programs launched as part of their national public health policies (8–11), whereas in others, including China, these programs, including colonoscopy, are currently being implemented. Therefore, examining the effectiveness of these screening programs is critical. Actual screening and medical insurance at a from an eastern Chinese city were examined to determine the need for screening, select the most appropriate screening strategy, and suggest methods to enhance screening implementation.

## 2 Materials and methods

### 2.1 Study design

A social perspective was selected as the research perspective, a model-based design was used, and residents of Huzhou City aged 50–75 years were identified as the target population. Based on the CRC screening project for key populations, 804,180 residents of Huzhou City were estimated to be aged 50–75 years. During the modeling phase, the population was set at 804,180 individuals, and the time horizon was set at 10 years. The incremental cost-effectiveness ratios (ICERs) were calculated to determine the value of each screening method, and the optimal screening strategy was determined by comparing the ICERs. The variability and uncertainty were assessed using one-way sensitivity, probabilistic sensitivity, and scenario analyses.

### 2.2 Ethics approval and consent to participate

Ethical considerations were in accordance with the Declaration of Helsinki. The study protocol was reviewed and approved by the Institutional Review Board approval # 2022-12-1019, and was also registered internationally (IRB00002408 & FWA00002399).

### 2.3 Setting and location

This study was conducted in Huzhou City, Zhejiang Province, China. The total population of Huzhou is 2.68 million, with the Han ethnic group accounting for 96.57% of the population. The overall sex ratio (calculated as the number of males per 100 females) is 109.04 and 18.70% of the population is aged 60 years or above. The per capita gross domestic product (GDP) of Huzhou is approximately 17,324 United States dollars (USD). The number of healthcare workers in Huzhou is approximately 11.74 per 1,000 people, and the number of hospital beds is approximately 6.37 per 1,000 people. According to data from 14 cancer registries in Zhejiang Province, the incidence rate of CRC is approximately 41.75 per 100,000 people and the mortality rate due to CRC is approximately 17.02 per 100,000 people.

### 2.4 Screening strategies

Our CRC screening strategy consisted of three approaches: no screening, sequential screening, and direct colonoscopy (1). No screening: No proactive measures are taken, and patients are diagnosed when they seek medical attention following symptom manifestation. (2) Sequential screening: This two-step process uses a two-sample FIT and risk assessment questionnaire. A positive result in the FIT (20μg of human hemoglobin per gram of stool, with samples taken 1 week apart) or a high-risk designation from the questionnaire leads to a recommendation for colonoscopy. (3) Direct colonoscopy: Individuals who undergo a colonoscopy without prior screening.

The risk assessment questionnaire included seven indicators: history of colorectal polyposis, familial adenomatous polyposis, age, sex, family history of CRC, smoking, and body mass index (**Table 1**). These were drawn from the Chinese CRC screening guidelines and validated through a meta-analysis. We further validated these indicators based on the detection results in our study.

**Table 1:** Risk evaluation questionnaire.

Factors	Group	Points	Risk
**History of colorectal polyposis**		5	≥ 5 points, High-risk 0-4 points, Low-risk
**Familial adenomatous polyposis**		5	
**Age (years)**	50-54	0	
	55-64	1	
	65-74	2	
**Gender**	Female	0	
	male	1	
**Family history of colorectal cancer**	No	0	
	Yes	1	
**Smoking**	Non-smoking	0	
	Smoking	1	
**Body mass index (kg/m2)**	<23	0	
	≥ 23	1	

### 2.5 Data sources

The CRC screening program began in 2019 and initially targeted a small subset of the population. In 2020, screening was officially launched with the aim of selecting one-fifth of the local target population (aged 45–74 years) annually. Within a 5-year interval, individuals within the target population will not be duplicated; however, new participants who meet the age criterion may still be included. Beginning in 2020, data from all individuals became accessible for analysis, and this study used data from 2020 to 2022. Owing to the large scale of the data, the electronic entry and verification processes exceeded 1year, making the 2022 data the most recently available. Participants were recruited from community hospitals and rural health service centers by doctors who typically invited residents to participate in the screening through posted flyers and phone notifications. This study employed a model-based approach with parameters derived from the following primary sources. The Huzhou Key Population Colorectal Cancer Screening Project was critical to our dataset, as it incorporates a two-step sequential screening strategy (**Additional File 1** in **Supplementary Material**). Through this project, we obtained acceptance rates for preliminary screening and subsequent colonoscopy-based evaluations, diagnostic results for the participants, and the costs associated with the initial screening. Data from the Health Insurance Bureau provided insights into treatment expenses related to early-stage CRC, advanced CRC, and metastatic advanced CRC, along with colonoscopy fees. Data from the Seventh Population Census were used to establish life tables (**Additional File 2** in **Supplementary Material**). Additionally, information from the Huzhou Municipal Bureau of Statistics contributed to our understanding of Huzhou’s GDP and other foundational demographic and infrastructural data. For parameters not readily available from the aforementioned data sources, we relied on data from relevant literature (**Table 2**).

**Table 2:** Model parameters and data sources.

Variable	Value	Distribution		Source
		type	Range	
Status distribution				
Low-risk population	0.797	Beta	0.641 to 0.953	CRCS
High-risk population	0.203	Beta	0.163 to 0.243	CRCS
CRC in general Population	0.0099	Beta	0.0080 to 0.0118	CRCS
CRC in low-risk population	0.0055	Beta	0.0046 to 0.0066	CRCS
CRC in high-risk population	0.027	Beta	0.0217 to 0.0323	CRCS
Utilities (Health effectiveness)				
Death	0	–	–	–
Health	1	–	–	–
Early CRC	0.83	Beta	0.747 to 0.913	Ref (25, 26)
Adv-CRC without metastasis	0.66	Beta	0.594 to 0.726	Ref (25, 26)
Adv-CRC with metastasis	0.54	Beta	0.486 to 0.594	Ref (25, 26)
Screening- diagnosed CRC	0.85	Beta	0.68 to 0.91	Stage-weighted ^b^
Self – referred CRC	0.74	Beta	0.60 to 0.89	Stage-weighted ^b^
Cost (USD/per subject)				
Sequential Preliminary screening	5	Gamma	4 to 6	CRCS
Colonoscopy	195	Gamma	157 to 233	HSA
Early CRC case	3249	Gamma	2,612 to 3,886	HSA
Adv-CRC without metastasis	7892	Gamma	6,346 to 9,439	HSA
Adv-CRC with metastasis	14034	Gamma	11,283 to 16,784	HSA
Per-capita income (daily)	37	Gamma	30 to 45	BS
Screening-diagnosed CRC	6286	Gamma	6,118 to 9,062	Stage-weighted ^b^
Hospital-diagnosed CRC	11672	Gamma	11,306 to 16,748	Stage-weighted ^b^
Cure rate a				
Early CRC	0.84	Beta	0.756 to 0.924	Ref (23)
Adv-CRC without metastasis	0.64	Beta	0.576 to 0.704	Ref (23)
Adv-CRC with metastasis	0.09	Beta	0.081 to 0.099	Ref (23)
Screening- diagnosed CRC	0.77	Beta	0.62 to 0.92	Stage-weighted ^b^
Self – referred CRC	0.54	Beta	0.43 to 0.65	Stage-weighted ^b^
Incidence rate				
Health to CRC (General population)	0.002	Beta	0.0016 to 0.0024	Ref (21)
Health to CRC (Post-colonoscopy population)				
5 years	0.00020	Beta	0.00012 to 0.00023	Ref (22)
10 years	0.00035	Beta	0.00021 to 0.00041	
Other parameters				
Sensitivity of sequential primary screening	0.83	Beta	0.65 to 0.95	Ref (18)
Discount rate per year	0.05	Beta	0.3 to 0.8	Ref (28)
Population mortality	0.0063	Beta	0.0051 to 0.0075	Age-weighted ^c^
GDP (USD)	17,324	Gamma	14,096 to 20,881	BS

Adv-CRC, advanced colorectal cancer; BS, Huzhou Municipal Bureau of Statistics; CRC, colorectal cancer; CRCS project, colorectal cancer screening project for key populations in Zhejiang Province; USD, United States Dollar; GDP, Gross domestic production; HAS, Health Care Security Administration; Self-referred CRCs are colorectal cancer patients who sought medical services without a screening. ^a^ cure rates were weighted according to age distribution in the model; ^b^ Including direct medical costs, direct non-medical costs, and indirect costs; ^c^ obtained via age-weighting following the creation of the life table.

### 2.6 Health status distribution

In the model, the health status of individuals was classified into three categories: non-CRC, CRC, and death. Among the patients who underwent sequential screening, 20.3% were classified as high-risk and 79.0% as low-risk based on primary screening. Of the 15,019 high-risk individuals who underwent colonoscopy, 411 (2.7%) were diagnosed with CRC. The probability of developing CRC in high-risk individuals who refused colonoscopy was assumed to be the same as that in individuals who underwent colonoscopy. The sensitivity of sequential primary screening was set at 83% based on a meta-analysis of 40 publications on individuals screened for CRC according to the Chinese screening guidelines, which reported a sensitivity of 83% for the FIT (18).Consequently, the probability of CRC in the low-risk population was expected to be 0.6%, resulting in an estimated 1.0% (8,042/804,180) in the same age range (50–75 years) in the Huzhou natural population. Early CRC was defined as cancer limited to the mucosa or invading the submucosa only (19, 20). Advanced CRC was defined as cancer that had penetrated the submucosa with metastasis or local progression. Of the 411 patients in the screening population diagnosed with CRC, 269 (65.5%) had early-stage (stage I) tumors, and 142 (34.5%)had advanced-stage (stages II–IV) tumors, with none having metastases (stage IV). In comparison, of the 10,150 self-referred patients with CRC, 433 (4.3%) had early-stage CRC and 9,717 (95.7%) had advanced-stage tumors. Although the databases did not report metastases, the rate was estimated to be 20% based on the results of other studies. The cancer stage was used in the model to determine weight costs and healthcare outcomes.

### 2.7 Incidence

The incidence of CRC in the general population has been reported to be 0.002 and decreases following colonoscopy (21, 22). The standardized incidence rates of CRC were 19.77 per 100,000 person-years 0–5 years after colonoscopy and 35.21 per 100,000 person-years 5–10 years after colonoscopy (22).

### 2.8 Cure and mortality rates

The duration for the decision-tree Markov model was set at 10 years. However, patients with CRC were not followed up; rather, data from a study that included a similarly aged population from similar regions with a 10-year follow-up period were used (23). In the present study, survival at 10 years was considered a cure. The 10-year survival rates of patients with CRC stages I–IV were 83.8%, 70.1%, 57.3%, and 8.7%, respectively. The stage-weighted cure rates were 76.9% in patients with CRC detected through screening and 53.6% in self-referred patients with CRC. The 1-, 3-, 5-, and 10-year stage-weighted CRC death rates were 2.4%, 7.8%, 13.1%, and 23.1%, respectively, among the screened patients, and 9.2%, 24.9%, 34.8%, and 46.4%, respectively, among those who were self-referred.

### 2.9 Cost determination

Costs included direct and indirect costs. Screening costs included 5 USD for the risk questionnaire and FIT and 195 USD for the colonoscopy. The total costs hospitalization costs for patients with early and advanced-stage CRC, with and without metastases, were calculated based on the medical insurance records of Huzhou City in 2020. The mean ± standard deviation (SD) direct costs to patients with early-stage CRC were 3249 ± 3,932 USD. In contrast, the mean ± SD direct costs to patients with advanced-stage CRC comprised 1,751 ± 1,831USD for hospitalization and 1,024 ± 742 USD for adjuvant treatment, the latter of which was administered six times to patients without metastasis and 12 times to patients with metastasis. Direct nonmedical and indirect costs were based on productivity costs. These costs were calculated based on the length of hospital stay, costs to the patient and one caregiver, and the per capita income in Huzhou. The average length of hospitalization was 11.7 ± 10.1 days for patients with early CRC In contrast, for patients with advanced CRC, the average hospitalization and adjuvant treatment lengths were 11.8 ± 12.0 days and 3.6 ± 3.7 days, respectively. In 2020, employees in Huzhou City earned an average of 37 USD per day. In accordance with the China Guidelines for Pharmacoeconomic Evaluations, the cost discount was set at 5% and the sensitivity analysis ranged from 0%–8% (24). The same discount rate was applied to the health outcome index. To simplify the model, all CRC stages were included and weighted in different populations. After weighting, the mean cost for individuals diagnosed with CRC through screening was set at 6,286 USD, whereas the mean cost for individuals diagnosed through self-referred visits was set at 11,672 USD.

### 2.10 Health outcomes

Health outcomes were determined by assessing the quality of life of patients with different stages of CRC in the same regions. These outcomes were based on the EuroQol five-dimension five-level index scores, in which the health outcomes of patients with CRC stages I–IV were set at 0.893, 0.821, 0.698, and 0.637, respectively, weighted according to the proportion of patients with these CRC stages in different populations (25). The stage-weighted CRC health effectiveness was set at 0.85 for patients diagnosed by screening and 0.74 for self-referred patients. The incremental cost, quality-adjusted life years (QALYs), and ICER were calculated.

### 2.11 Model

**Figure 1:**
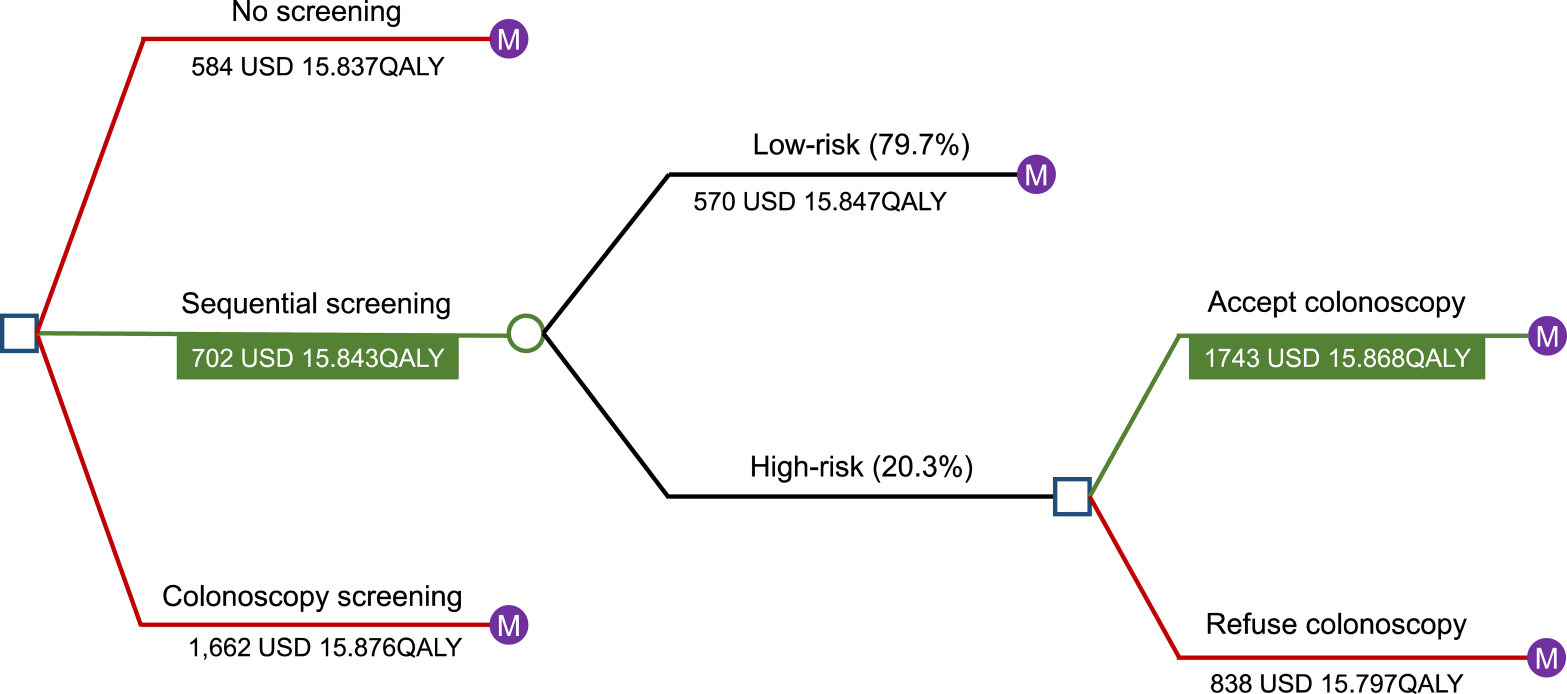
Results of the Markov decision tree analysis. A decision-tree Markov model was established to estimate the impact of different colorectal screening strategies on disease burden, QALYs, and costs for individuals aged 50–75 years in Huzhou City. Individuals in the cohort were divided into three categories: healthy, CRC, and deceased. A decision tree was created at the beginning of the model, with participants free to choose whether to undergo screening. Participants were allowed to enter in any state and could transition to death from any health state. Sequential primary screening could provide positive or negative results, and based on probability, individuals with positive primary screening results were assigned to undergo or refuse colonoscopy. The model compared the ICER to the target population’s decision to initiate screening or not, as well as to various screening strategies. Abbreviations: USD, United States dollar; QALYs, quality-adjusted life years; CRC, colorectal cancer; ICER, incremental cost-effectiveness ratio.

A decision-tree Markov model was established to estimate the impact of different colorectal screening strategies on disease burden, QALYs, and costs for individuals aged 50–75 years in Huzhou City (**Figure 1**). A decision tree was developed at the beginning of the model, with individuals free to choose whether to undergo screening. The participants were allowed to enter in any state and could transition to death from any health state. Sequential primary screening could provide positive or negative results; based on this probability, individuals with positive primary screening results were assigned to undergo or refuse colonoscopy. The model compared the ICER to the target population’s decision to initiate screening or not, as well as to various screening strategies. Individuals in the cohort were divided into three categories: healthy, CRC, and deceased. The probability of a person with a given set of primary screening results being in a category was determined based on the results of the screening project. Statistical analyses were performed using TreeAge Pro Healthcare 2020 software (TreeAge Pro 2020, R1. TreeAge Software, Williamstown, MA, USA) and R Statistical Software (version 4.2.1; R Foundation for Statistical Computing, Vienna, Austria). A checklist for this guidance is provided in **Additional File 4** in **Supplementary Material**.

## 3 Results

This study included 175,550 individuals who underwent screening; of these, 35,555 (20.3%) were evaluated as high-risk. Of these high-risk individuals, 15,019 (42.2%) underwent colonoscopy, of whom, 411 (2.7%) were diagnosed with CRC. Colonoscopy acceptance rates were associated with the questionnaire scores and the number of FIT-positive results. Among individuals with a high risk, those with 0, 1, and 2 FIT positive results had colonoscopy acceptance rates of 26.3% (3,007/11,449), 41.3% (1,181/2,859), and 65.7% (435/662), respectively. Among individuals with questionnaire scores of 0-4 points, those with one and two FIT-positive results had colonoscopy acceptance rates of 46.8% (7,332/15,665) and 62.3% (3,064/4,920), respectively. The colonoscopy acceptance rate was primarily affected by the number of FIT positive results. Patients who were divided into questionnaire-positive(5 points), FIT-positive, and doubly-positive on primary screening had early CRC rates of 83.3% (30/36), 64.1% (207/323), and 61.5% (32/52), respectively, with the proportion of early-stage tumors being higher in the questionnaire-positive group than in the FIT-positive (P _questionnaire_ vs. P _FIT_ =0.033) and doubly positive (P _questionnaire_ vs. P _questionnaire + FIT_=0.049) groups.

Screening benefits were evaluated in different situations. The ICER showed that sequential screening was more cost-effective (**Table 3**, **Figure 1**). According to situation 1 (actual results of the CRC screening project in key populations), only 8.6% of the entire population underwent colonoscopy, resulting in the avoidance of 292 (13.6%) deaths from CRC, whereas colonoscopy screening would have prevented 160 (7.5%) CRC deaths. According to situation 2 (ideal sequential screening), 691 (32.2%) deaths from CRC would be avoided, whereas the same colonoscopy acceptance rate in patients screened by colonoscopy would prevent 377 (17.6%) deaths from CRC. To reduce the number of CRC deaths to the same level as that in situation 2, the colonoscopy acceptance rate in individuals screened by colonoscopy would have to increase to 37.2%.

**Table 3:** Results of various screening situations.

Result	General		Acceptance rate of colonoscopy screening			Sequential screening	
		100%	37.2%	20.30%	8.6%	Situation 1^a^	Situation 2 ^b^
New CRC	15539	2017	10509	20746	14376	14387	12808
Cum CRC deaths	2147	290	1456	1770	1987	1855	1456
Cum Eff	15.837	15.876	15.851	15.845	15.840	15.843	15.851
Cum cost	584	1662	985	803	677	702	808
InEff		0.039	0.014	0.008	0.003	0.006	0.014
InCost		1078	401	219	93	118	224
ICER			27379			19335	15516

^a^ Situation 1 is the real situation for sequential screening, in which 20.3% of the population is classified as high risk and 42.2% of them completed colonoscopy (The number of completed colonoscopies was 8.6% of the total population.); ^b^ Situation 2 is the optimal situation for sequential screening, in which all high-risk individuals have colonoscopy; CRC, colorectal cancer; Cum, Cumulative; Eff, Effectiveness;In, Incremental;ICER, Incremental Cost Effectiveness Ratio.

**Figure 2:**
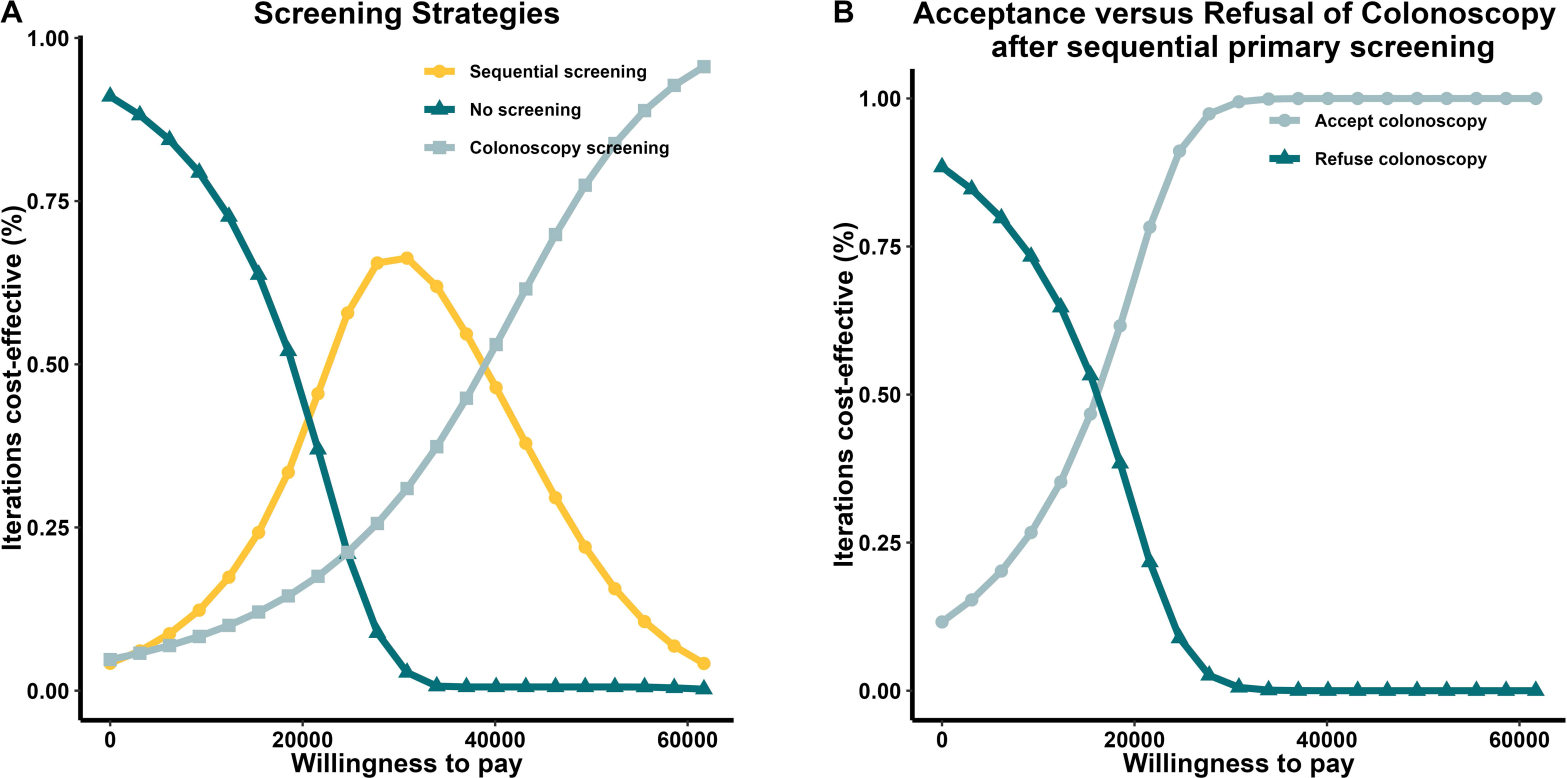
Tornado diagram. A one-way sensitivity analysis of the four decisions included in this study, using the ICER as the criterion, enabled the selection of the five factors with the greatest influence on each outcome. The resulting tornado diagram showed that the cost of colonoscopy had a major impact on individuals who underwent screening. The sensitivity of sequential primary screening had the greatest impact on the choice between sequential and colonoscopy screenings. Abbreviations: EV, economic value; ICER, incremental cost-effectiveness ratio.

The one-way sensitivity analysis of the four decisions, using the ICER as the criterion enabled the selection of the five factors with the greatest influence on each outcome (**Figure 2**).The resulting tornado diagram shows the cost of colonoscopy had a major impact on individuals who underwent screening. The sensitivity of sequential primary screening had the greatest impact on the choice between sequential and colonoscopy screenings. In patients with positive initial screening results, the cost of colonoscopy affects the decision to continue with the procedure. The incremental cost-effectiveness scatter plot demonstrates that sequential and direct colonoscopy screening is superior to no screening at the current threshold. Notably, individuals who test positive during the initial sequential screening should be referred for colonoscopy. The probabilistic sensitivity analysis results are reported in **Additional File 3** in **Supplementary Material**.

**Figure 3:**
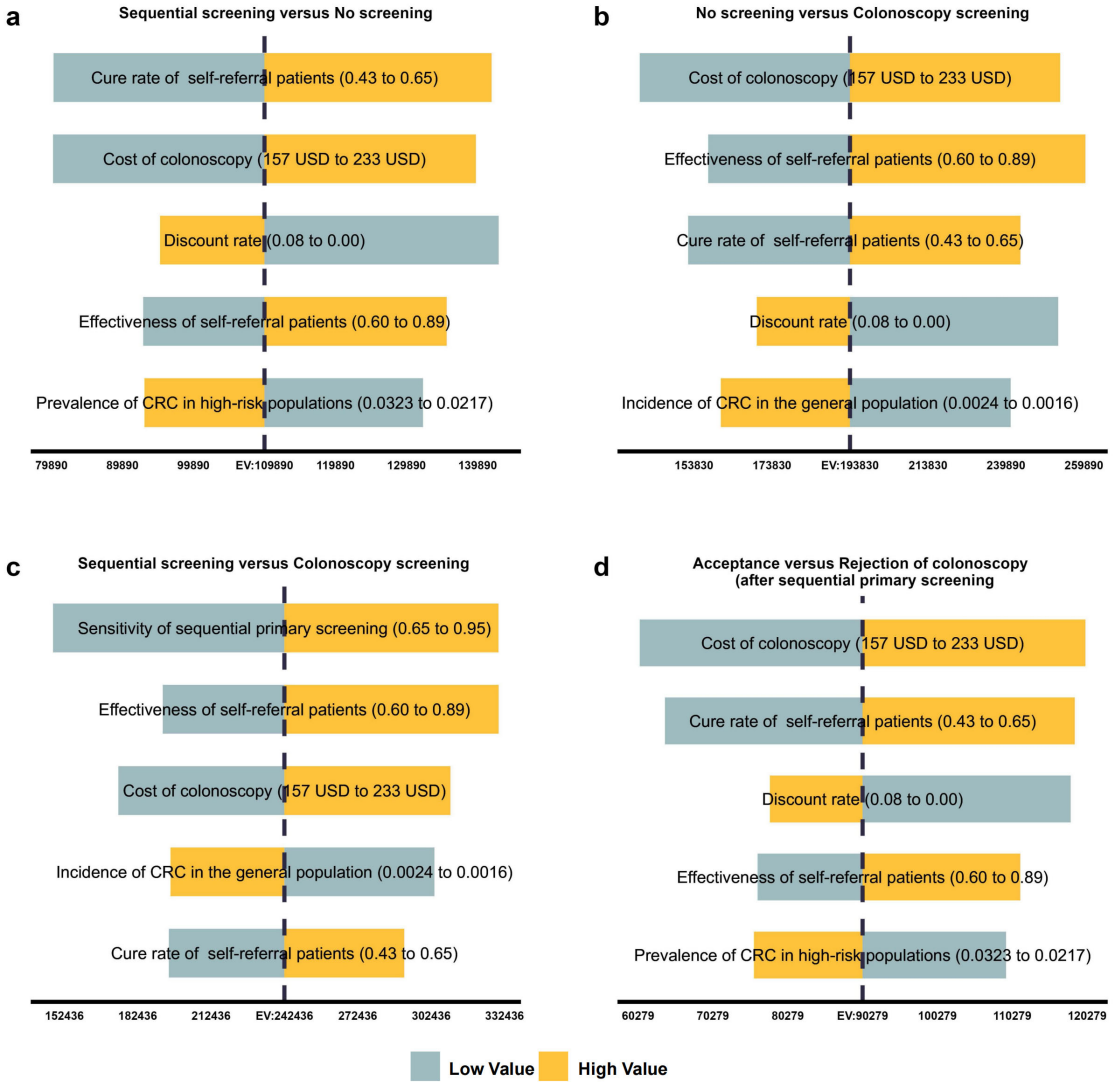
Cost-effectiveness acceptability curve. Results of the CE acceptance curves and the per capita (GDP) of 17,324 USD showed that when the WTP was within approximately one to two times the per capita GDP range, sequential primary screening was preferred. In contrast, when the WTP was greater than two times the per capita GDP, colonoscopy screening was more likely to be chosen. Comparisons of acceptance versus refusal after sequential primary screening showed that when the WTP was close to the per capita GDP, individuals were more likely to proceed to colonoscopy, the second step of sequential screening. CE, cost-effectiveness; GDP, gross domestic product; USD, United States dollar; WTP, willingness-to-pay.

The cost-effectiveness acceptance curves and the per capita GDP of 17,324USD showed that sequential primary screening was preferred when the willingness-to-pay (WTP) was within approximately one to two times the per capita GDP range. In contrast, colonoscopy screening was more likely to be chosen when the WTP was greater than two times the per capita GDP. Comparisons of acceptance versus refusal after sequential primary screening showed that, when the WTP was close to the per capita GDP, individuals were more likely to proceed to colonoscopy, the second step of sequential screening (**Figure 3**).

## 4 Discussion

By integrating a substantial volume of screening and medical insurance data, we employed a decision-tree Markov model to comprehensively assess the cost-effectiveness of three CRC screening strategies: no screening, sequential two-step screening, and colonoscopy screening. The findings revealed that colonoscopy and initial sequential screening were cost-effective. According to the ICER criterion, sequential screening is the preferred option, and it is essential to underscore the existence of an upper limit to the health benefits achievable through a two-step screening approach. Therefore, this approach is particularly suitable for the initial phases of screening implementation. With increased screening duration and higher acceptance rates among the population, transitioning to direct colonoscopy screening is recommended. One of the primary considerations for enhancing the individual acceptance rates of colonoscopy is the reduction in colonoscopy costs.

The main health benefit of screening is the early diagnosis of CRC. The data from this study show that the proportion of early-stage patients identified through screening was higher than that of self-referred patients. Early diagnosis resulted in a cure rate over nine times higher than in patients with metastases but also to provide a superior quality of life (23, 25, 26). In addition, early diagnosis resulted in a shorter treatment period, with accumulated hospital stays of approximately 11.7 days in early-stage patients, 33.4 days in advanced-stage patients without metastases, and approximately 55 days in advanced-stage patients with metastases. The costs of early diagnosis are also lower, with medical insurance data showing that the costs in early-stage patients were approximately 41.2% of those in advanced-stage patients without metastases and 23.2% of those in advanced-stage patients with metastases.

Nationwide colonoscopy screening programs for CRC have been introduced in developed regions such as Europe and the United States (8–11). Screening reduces the incidence and mortality of CRC (16). More recently, however, the effectiveness of colonoscopy has been questioned, with screening being found to be ineffective in reducing the incidence and mortality of CRC (17). This study suggests that the acceptance rate of colonoscopy may be the main factor affecting the benefits of screening. In this study, only 42% of the participants in the sequential screening group underwent colonoscopy. If individuals in the screening group who refused colonoscopy were excluded, the incidence and death rates of CRC would have been reduced by 31% and 50%, respectively (17). Similar outcomes were obtained in the present study, indicating that the acceptance rate of colonoscopy substantially affects screening effectiveness. Based on a questionnaire assessment, only 26.3% of individuals in the FIT-negative but high-risk group underwent colonoscopy, which may be even lower in the general population. Similar studies in China may find that the colonoscopy acceptance rate is below 26.3%, indicating that colonoscopy screening alone may not be suitable for developing countries preparing to implement new screening strategies.

Sequential screening was defined as a low-cost, non-invasive primary screening, consisting of risk questionnaires and FITs, followed by colonoscopy in high-risk individuals. This strategy allows high-risk individuals to undergo colonoscopy at the same cost, making it more cost-effective from social and healthcare system perspectives. Owing to the increasing incidence of CRC in developing nations (27), this cost-effective screening strategy should be widely promoted.

The present study also found that the results of sequential primary screening can affect individuals’ willingness to undergo a second-step colonoscopy. Therefore, in nations that have implemented colonoscopy screening programs, primary screening of those unwilling to undergo colonoscopy may directly increase the acceptance rate. This strategy can improve the colonoscopy acceptance rate in countries where this rate has stabilized and is no longer increasing. The sensitivity analysis results also suggest the future use of more sensitive primary screening methods (18). Although the high-risk questionnaire did not increase patient acceptance of colonoscopy, it effectively identified more patients with early-stage CRC. Improving and promoting the questionnaire may increase screening acceptance rates.

The present study has several limitations. Because we chose some parameters from the literature and despite our efforts to select high-quality research and consider the study areas and target populations, bias was inevitable. The screening data were obtained from records collected in Huzhou between 2020 and 2022. When selecting screening participants, we focused exclusively on residents of Huzhou aged 45–74 years to enhance the generalizability of the findings. However, selection bias was unavoidable, precluding the elimination of bias among the research participants. Individuals with confidence in their health or those declining participation in our screening because of other factors, such as regular health checkups at their workplace, might have still opted to forego the screening, even after being recruited. Second, although we analyzed the direct non-medical and indirect labor costs to patients and caregivers resulting from patient hospitalization, we omitted other costs, including food and accommodation, transportation, nutrition, and informal care. Thus, the costs of CRC and the cost-effectiveness of screening might have been underestimated. Third, the potential risks of colonoscopy are among the reasons individuals resist this procedure. In this study, only two of the 15,019 patients who underwent colonoscopy experienced moderate bleeding, and none experienced serious complications. However, the model did not account for these events, which might have resulted in an overestimation of the health advantages in the screening group. Finally, because the CRC incidence rate in the low-risk groups could not be obtained during the modeling stage, it was replaced by the incidence rate in the general population. This could have overestimated the incidence of CRC diagnoses and deaths in the sequential screening group while underestimating the health benefits.

In developing nations, CRC screening is still in its infancy. Future studies should include additional data to enhance the described model and provide more precise results. Individual-based microsimulation models are also under development. Future studies should include additional characteristics resulting in more accurate analyses of the cost-effectiveness of screening and the development of more effective screening strategies.

## 5 Conclusions

We evaluated the cost-effectiveness of three CRC screening strategies: no screening, sequential two-step screening, and colonoscopy. Sequential screening is more cost-effective than colonoscopy screening, especially in regions with limited resources or low colonoscopy acceptance rates. However, colonoscopy screening is more advantageous than sequential screening in preventing CRC deaths when costs are not a barrier. Therefore, we recommend sequential screening as a feasible and effective approach for individuals residing in regions with limited resources or in the initial phases of implementing regional screening programs. In areas where regional colonoscopy screening has been implemented, utilizing the FIT for preliminary assessment can significantly improve the acceptance rates of colonoscopy in individuals who decline direct colonoscopy, thus effectively increasing the colonoscopy acceptance rate.

## Data Availability

The raw data supporting the conclusions of this article will be made available by the authors, without undue reservation.
