# Supplementary material for: Cost-effectiveness analysis of sequential two-step screening versus direct colonoscopy screening for colorectal cancer: a large-scale survey in Eastern China
Source: Front Oncol. 2025 Feb 14;15:1524172. doi: 10.3389/fonc.2025.1524172 (PMC11867945; doi:10.3389/fonc.2025.1524172)
Supplement: Supplementary file 1 [file DataSheet1.docx]

## Additional file 2: Life table

**Additional table 2 Life table^a^**

| Age | Mortality(m_x_) | Probability of dying(q_x_) | Expectations of life (e_x_) |
| --- | --- | --- | --- |
| 50~ | 0.0017 | 0.0084 | 35.0 |
| 55~ | 0.0028 | 0.0137 | 30.2 |
| 60~ | 0.0053 | 0.0260 | 25.6 |
| 65~ | 0.0081 | 0.0397 | 21.2 |
| 70~ | 0.0142 | 0.0684 | 17.0 |
| 75~ | 0.0275 | 0.1289 | 13.1 |

^a^ Due to the paucity of data from the Huzhou City census, we have chosen to build the life tables using data from the Zhejiang Province census of 2020. (Huzhou City is part of Zhejiang Province).
