# Supplementary material for: Cost-effectiveness analysis of sequential two-step screening versus direct colonoscopy screening for colorectal cancer: a large-scale survey in Eastern China
Source: Front Oncol. 2025 Feb 14;15:1524172. doi: 10.3389/fonc.2025.1524172 (PMC11867945; doi:10.3389/fonc.2025.1524172)
Supplement: Supplementary file 2 [file DataSheet2.docx]

## Additional file 3: Incremental Cost-Effectiveness Scatter Plot


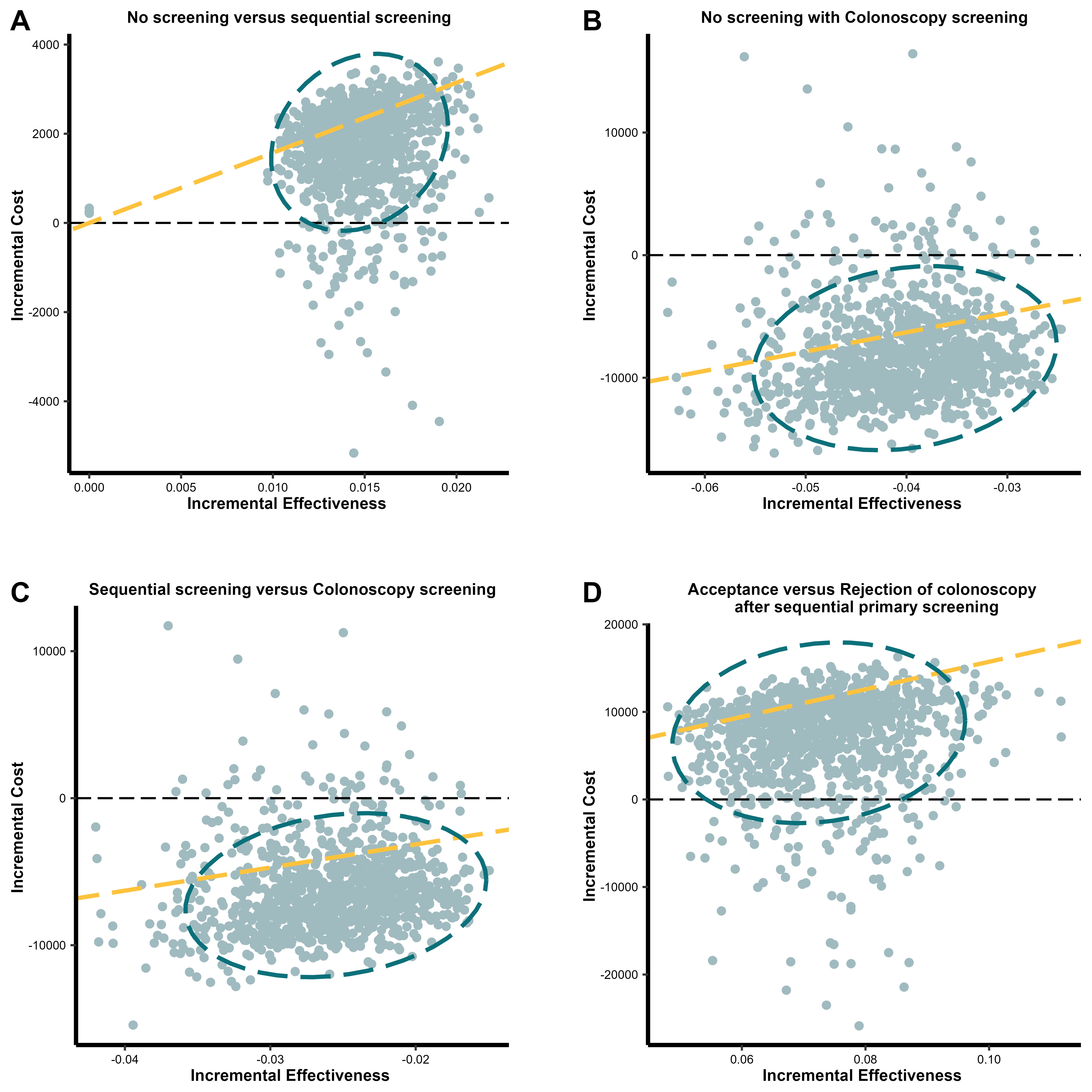


Figure legend: The probabilistic sensitivity analysis of the four decisions included in this study, using curves and the per capita (GDP) as the criterion.
