# Supplementary figures and images for: Cost-effectiveness analysis of sequential two-step screening versus direct colonoscopy screening for colorectal cancer: a large-scale survey in Eastern China
Source: Front Oncol. 2025 Feb 14;15:1524172. doi: 10.3389/fonc.2025.1524172 (PMC11867945; doi:10.3389/fonc.2025.1524172)

## Additional file 1: Process of Huzhou Key Population CRC Screening Project


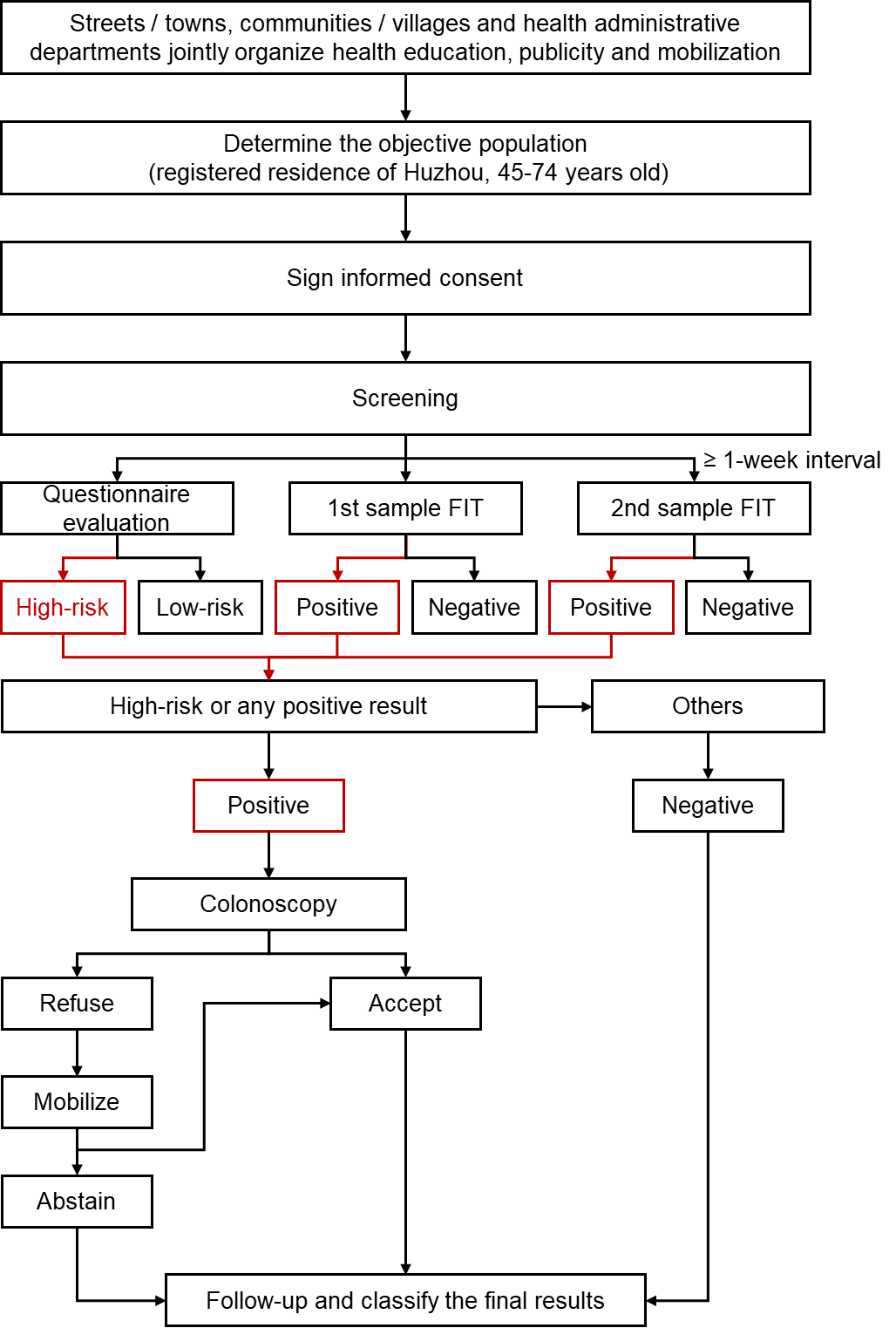

Supplement: Supplementary file 3 [file DataSheet3.docx]
